# Gustatory Detection of Tetrodotoxin and Saxitoxin, and Its Competitive Inhibition by Quinine and Strychnine in Freshwater Fishes

**DOI:** 10.3390/md9112283

**Published:** 2011-11-08

**Authors:** Toshiaki J. Hara

**Affiliations:** Department of Biological Sciences, University of Manitoba, Winnipeg, Manitoba, R3T 2N2, Canada; E-Mail: thara@cc.umanitoba.ca or t-hara@lime.plala.or.jp; Tel.: +1-1-204-983-5010; Fax: +1-81-96-456-2555

**Keywords:** fish, gustation, tertrodotoxin, quinine, strychnine, inhibition, antidote

## Abstract

Fish detect extremely low levels of marine toxins tetrodotoxin (TTX) and saxitoxin (STX) via the specialized gustatory receptor(s). Physiological and pharmacological studies show that receptor(s) for TTX and STX are distinct from those which detect feeding stimulant amino acids and bile acids, and that TTX and STX do not share the same receptor populations, while interacting with quinine and strychnine in a competitive fashion suggestive of an antidotal relationship.

## 1. Introduction

Fish, like all other vertebrates, detect chemical stimuli primarily through olfaction and gustation. The function of chemical senses is the most ancient of sensory systems, evolved 500 million years ago, and mediate the animals’ function most basic to the survival of the individuals and species: feeding and reproduction. The aquatic environment surrounding fishes makes their chemical senses unique, and distinction between these two sensory modalities is not always as clear as in terrestrial organisms. In air or water, however, all chemoreception ultimately is an aquatic phenomenon, because the receptors are covered with fluid materials where the initial transduction process takes place [1].

Fish, constituting more than half of the total number of *ca.* 50,000 recognized living vertebrate species, are a pivotal group that has served as a model for the study of the vertebrate chemosensory system. The fishes often possess specific, sensitive and readily accessible chemosensory organs, which has permitted a critical evaluation of the several transduction sequences that follows the initial receptor binding step [2]. The logic of the most enigmatic of sensory system, and homologous families of olfactory receptor genes have now been identified in a variety of vertebrate species including fishes [3,4]. Phylogenetic analyses of olfactory receptor genes suggest that the most recent common ancestor between fishes and tetrapods had at least nine ancestral olfactory receptor genes, and all olfactory receptor genes identified are classified into nine groups, each of which originated from one ancestral gene [5].

Gustatory system, by contrast, has evolved reflecting their diverse modes of lives in the aquatic environment. Hypertrophy of the system has occurred independently in several groups, notably in the siluroids and the cyprinids. The gustatory system has traditionally been thought to be the primary channel for the detection of chemical cues for feeding. However, recent studies demonstrate that in many fish species feeding is triggered primarily through olfaction, complemented by gustation [6–8]. Fish gustatory receptors are generally highly sensitive to alkaloids such as quinine (Q·HCl) and strychnine, and there is growing evidence suggesting that the CO_2_ sensitivity of gustatory receptors might be involved in the ventilatory/respiratory regulation in fishes [7,9,10]. Of particular interest is the extreme gustatory sensitivity to bile acids and marine neurotoxins including tetrodotoxin (TTX) and saxitoxin (STX), and their receptor interactions. Therefore, the statement such as, “Where studied, gustation in fish appears to be exclusively associated with feeding.” may no longer be valid.

The purpose of this review is to summarize the current issues in fish chemoreception, with particular emphasis on the extreme gustatory sensitivity to alkaloids and marine toxins. The evidence is also be presented indicating that bile acids, the most potent gustatory stimulants recorded, are detected via a specific receptor type independent of those for feeding stimulant amino acids, and that TTX, quinine and strychnine may partially share the same receptor mechanism.

## 2. Characteristics of Fish Olfaction and Gustation

Generally, olfaction in vertebrates is a distance chemical sense with high sensitivity and specificity, whereas gustation is primarily a contact or close-range sense with moderate sensitivity. However, the fish chemical sense is unique in that gustatory receptors are equally sensitive to the same chemical stimuli or even more sensitive than olfactory, making the distinction between the two modalities blurred. Thus, in fish solubility, rather than volatility, of chemical compounds determine their capacity as chemical cues, and consequently, nonvolatile compounds with relatively low molecular weights are superior substances to fish chemoreception.

**Figure 1:**
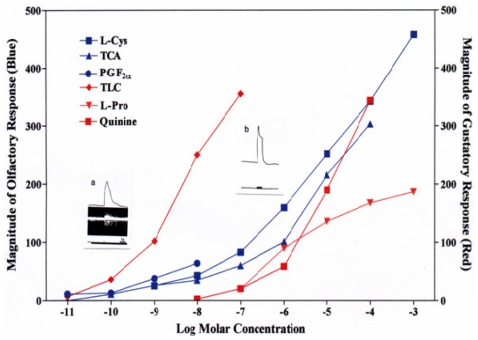
Comparison of the olfactory (electro-olfactogram, EOG) and gustatory (palatine nerve activity) sensitivities to representative chemical stimuli examined electrophysiologically in rainbow trout. Adapted with permission from Academic Press [8].

In olfaction, four main classes of chemical compounds have generally been identified as specific olfactory stimuli (odorants/pheromones) for many fish species and their stimulatory effectiveness characterized: amino acids, bile acids, sex steroids, and prostaglandins (Figure 1) [11,12]. These four odorant classes, detected by separate receptor families, are typically non-odorous to humans. Various alcohols, amines, carboxylic acids, nucleotides, and aromatic hydrocarbon have also been found to have olfactory activity in some fish species, but our understanding of their function is unclear [7,13,14].

Phylogenetic analyses of olfactory receptor genes indicate that the sizes of the receptor repertoires of vertebrate species are extremely large, and approximately 1% of all genes are devoted to smell, perhaps reflecting the significance of this sensory system for the survival of most vertebrate species, including fishes. Of these, amino acids are by far the most widely studied chemicals in fish olfaction. The olfactory spectrum of amino acids is generally similar across all fish species examined. All lines of experimental evidence indicate that multiple receptor types exist, which play dominant roles in discrimination of amino acids. The cloned goldfish olfactory receptor 5.24, for example, is preferentially tuned to recognize basic amino acids, arginine (Arg) and lysine, suggesting that this receptor may in fact represent an Arg receptor in this species [15]. Amino acids initiate early phases of feeding behavior (arousal and search), while prostaglandins are released into the water, where they function as pheromones that trigger spawning behaviors, e.g., digging gravel beds by female salmonids. Experimental results further provide evidence for the existence of the two functional olfactory subsystems in fish, one for odorants and the other for pheromones. Pheromones are processed in a manner distinct from those for odorants such as amino acids and bile acids, possibly mediated by extra-bulbar primary olfactory fibers bypassing the olfactory bulb [16–18].

In gustation, in which taste buds ontogenetically and phylogenetically develop later than their counterpart olfactory system, constitute the structural basis of the peripheral gustatory organ. Response spectra for amino acids vary considerably among fish species, contrasting with relatively consistent spectra of the olfactory described above. Roughly two groups are identified: one that responds to a few amino acids, represented by salmonids, and one that responds to many amino acids, represented by channel catfish [7,8,10]. All species in the former respond well to l-proline (Pro), l-alanine (Ala) and a few other related amino acids. Channel catfish, by contrast, detect virtually all common amino acids by gustation, with the sensitivities higher (lower thresholds) than those of the olfactory counterpart [10]. Thus, in fishes in the former group Pro is the dominant amino acid, minimizing the redundancy between the gustatory and olfactory systems. In fishes in the latter, Ala and Arg are dominant and considerable overlaps exist between the two.

## 3. High Gustatory Sensitivity and Specificity to TTX and STX

**Figure 2:**
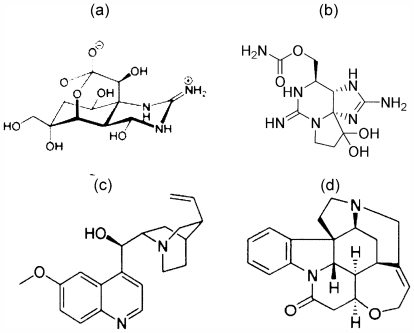
General chemical structures of (**a**) tetrodotoxin (TTX); (**b**) saxitoxin (STX); (**c**) quinine (Q·HCl); and (**d**) strychnine.

Contrary to what is generally held, fishes are able to avoid or reject unfavorable food items before being taken into the mouth cavity. Certain fish species show repulsive and avoidance reactions, or depressed locomotor and feeding activities to natural alkaloids quinine (Q·HCl) and strychnine, as well as puffer toxin tetrodotoxin (TTX) and shellfish toxin saxitoxin (STX) (Figure 2). Certain fish species show repulsive and avoidance reactions, or depressed locomotor and feeding activities to strychnine, Q·HCl and TTX. Q·HCl, for example, deters feeding in pufferfish, dover sole, and goldfish. Similarly, rainbow trout reject pieces of toxic pufferfish liver and artificial food pellets containing TTX.

TTX has been known as a popular chemical tool in the physiology and pharmacology communities since its discovery as Na-channel blocking action by Narahashi [19] in the early 1960s. STX is naturally produced by certain species of marine dinoflagellates, and also a neurotoxin that acts on the voltage-gated sodium channels of nerve cells. Q·HCl is best known for its anti-malarial and anti-inflammatory properties, and has a bitter taste and is a flavor component of tonic water and bitter lemon, while strychnine, a very toxic alkaloid used as a pesticide, is one of the most bitter substances known. Of particular interest here, however, is a characteristic functional behavior TTX and STX manifest in fish gustation.

**Figure 3:**
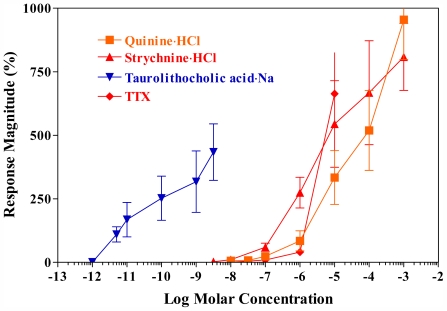
Concentration-response relationships of gustatory responses recorded from the rainbow trout palatine nerve bundles. TLCA, Taurolithocholic acid·Na. Adapted with permission from Academic Press [8].

High gustatory sensitivities to TTX and STX were first demonstrated in rainbow trout and Arctic char [20]. The threshold concentrations for both chemicals range between 10^−8^ and 10^−7^ M in rainbow trout (Figure 3). Cross-adaptation experiments indicate that the gustatory receptor(s) for TTX are distinct from those which detect amino acids and bile acids, and that TTX and STX do not share the same receptor populations, though both exert essentially the same effect on nerve membranes selectively blocking transient sodium channels [19]. This is in contrast with those of higher vertebrates where TTX does not stimulate the gustatory systems of frog and rat [21]. The result that perfusion of the palate (receptors) with 10^−6^ M TTX for more than 5 min had no effect on gustatory responses to amino acids suggest that the amino acid-activated TTX-sensitive cation channel(s) are not/is not present in the apical membrane of the gustatory cells and that TTX does not penetrate the tight junctions found at the top of the gustatory cells [20,22]. The sensitivity to TTX is unaffected by adaptation to STX, and vice versa. These results, combined with the fact that they have distinct C-R relationships, provide strong evidence that the receptor(s) for TTX are different from those for STX [23].

## 4. Competitive Inhibition of TTX Responses by Q·HCl, Strychnine and TLCA

**Figure 4:**
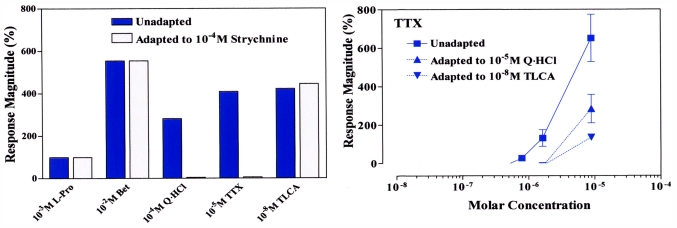
(**Left**) Interactions between strychnine, Q·HCl, bile acid (TLCA), and TTX in rainbow trout, demonstrating that Q·HCl and TTX are inhibited by strychnine, but L-Pro, betaine (Bet) and TLC are unaffected; (**Right**) TTX is suppressed by either Q·HCl (▴, 10^−5^ M) or TLCA (▾, 10^−8^ M ). Adapted with permission from Academic Press [8].

Pretreatment with strychnine in rainbow trout inhibits responses to Q·HCl and TTX in a competitive fashion, but without any effect on amino acid and bile acid responses, suggesting that all these chemicals share the same receptor and transduction mechanisms, despite vastly different molecular structures (Figure 4) [8,20,23]. The molecules consist of a positively charged guanidinium group and a pyrimidine ring with additional fused ring system (Figure 2). This situation would eventually create pseudo-competitive antagonists reversibly binding to receptors at the same binding site as the endogenous ligand or agonist, but without activating toxicity. Agonists (TTX) and antagonists (Q·HCl) or TLCA “compete” for the same binding site on the receptor. Once bound, an antagonist will replace agonist binding depending on their affinities, thus creating a possible antidotal function. Note that, in functional assays using competitive antagonists, a parallel rightward shift of agonist dose-response curves with no alteration of the maximal response is observed.

The TTX-sensitive, voltage-dependent, Na^+^ current is present in most gustatory cells [24]. In frog, the transient inward Na+ current in a patch-clamped gustatory cell is completely blocked by TTX [25]. However, in in vivo experiments, as described above, the generation of a receptor potential in response to gustatory stimuli is not inhibited by TTX [21]. Thus, the results with salmonids suggest that the l-Pro-activated TTX-sensitive cation channel(s) is not present in the apical membrane of the gustatory cells and that TTX does not penetrate the tight junctions found at the top of the gustatory cells.

## 5. Concluding Remarks

Fish detect extremely low levels of the marine toxins TTX and STX via the specialized gustatory receptor(s). Results from physiological and pharmacological studies are relevant to the interpretation that these receptors are distinct from those which detect other known gustatory stimuli including feeding stimulant amino acids and bile acids.

Ecologically, on one hand, evolution of the sensitive, specific receptor system for the toxins provides adaptive significance for predators; possible dual functions of the marine toxins as semiochemicals providing adaptive significance both to producing and receiving organisms. Toxicologically, on the other hand, strychnine and bile acid TLCA inhibit gustatory responses to TTX in a competitive fashion; strychnine is one of the most bitter (to human) and toxic substances known; TLCA, by contrast, is one of the most stimulatory, but a non-toxic substance in both olfactory and gustatory systems in fish, creating a unique counteracting situation. Further studies on their possible antidotal function are strongly suggested.
